# GLO1 cg26053840 Methylation Associates with Kidney Injury and Inflammatory Markers in Hospitalized Older Adults

**DOI:** 10.3390/life16060917

**Published:** 2026-05-29

**Authors:** Carlo Fortunato, Francesco Piacenza, Gretta Veronica Badillo Pazmay, Marco Malavolta, Maurizio Cardelli, Antonio Cherubini, Leonardo Biscetti, Giuseppe Pelliccioni, Luca Soraci, Davide Gentilini, Luciano Calzari, Francesca Marchegiani, Rina Recchioni, Chiara Giordani, Giulia Matacchione, Matilde Sbriscia, Sonia Fantone, Roberta Galeazzi, Fabrizia Lattanzio, Anna Rita Bonfigli, Mirko Di Rosa, Fabiola Olivieri, Robertina Giacconi

**Affiliations:** 1 Center of Biogerontology, IRCCS INRCA, 60124 Ancona, Italy; c.fortunato2@inrca.it (C.F.); f.piacenza@inrca.it (F.P.); g.badillo@inrca.it (G.V.B.P.); m.malavolta@inrca.it (M.M.); m.cardelli@inrca.it (M.C.); m.sbriscia@inrca.it (M.S.); s.fantone@inrca.it (S.F.); f.olivieri@staff.univpm.it (F.O.); 2 Department of Clinical and Molecular Sciences (DISCLIMO), Università Politecnica Delle Marche, 60131 Ancona, Italy; a.cherubini@inrca.it; 3 Geriatria, Accettazione Geriatrica e Centro di Ricerca per L’invecchiamento, IRCCS INRCA, 60124 Ancona, Italy; 4 Neurology Unit, IRCCS INRCA, 60124 Ancona, Italy; l.biscetti@inrca.it (L.B.); g.pelliccioni@inrca.it (G.P.); 5 Unit of Geriatric Medicine, IRCCS INRCA, 87100 Cosenza, Italy; l.soraci@inrca.it; 6 Department of Brain and Behavioral Sciences, University of Pavia, 27100 Pavia, Italy; davide.gentilini@unipv.it; 7 Bioinformatics and Statistical Genomics Unit, Istituto Auxologico Italiano IRCCS, Cusano Milanino, 20095 Milan, Italy; luciano.calza@gmail.com; 8 Clinic of Laboratory and Precision Medicine, IRCCS INRCA, 60124 Ancona, Italy; f.marchegiani2@inrca.it (F.M.); r.recchioni@inrca.it (R.R.); c.giordani@inrca.it (C.G.); g.matacchione@inrca.it (G.M.); r.galeazzi@inrca.it (R.G.); 9 Scientific Direction, IRCCS INRCA, 60124 Ancona, Italy; f.lattanzio@inrca.it (F.L.); a.bonfigli@inrca.it (A.R.B.); 10 Centre for Biostatistics and Applied Geriatric Clinical Epidemiology, IRCCS INRCA, 87100 Cosenza, Italy; m.dirosa@inrca.it

**Keywords:** GLO1, methylglyoxal, DNA methylation, hyperglycemia, inflammation, kidney dysfunction, aging

## Abstract

The glyoxalase pathway detoxifies reactive dicarbonyls generated during hyperglycemia, but the role of its epigenetic regulation in renal dysfunction and inflammatory dysregulation in older adults remains unclear. We investigated CpG-specific DNA methylation within the glyoxal detoxification pathway, focusing on the GLO1 gene, and examined associations with glycemic status, renal function, and systemic inflammation in hospitalized older adults. We identified a single CpG site within the GLO1 gene (cg26053840) significantly associated with fasting glycemia, suggesting that methylation levels at this locus reflects metabolic stress. Higher methylation at cg26053840 was also associated with impaired renal function, including increased serum creatinine and reduced estimated glomerular filtration rate. Additionally, GLO1 methylation correlated with multiple inflammatory indices, including C-reactive protein, erythrocyte sedimentation rate, neutrophil-to-lymphocyte ratio, and the CRP-to-albumin ratio. Associations with circulating cytokines and immune activation markers such as IL-6, IL-17A, GDF-15, CXCL9, CD163, and soluble RAGE further indicated broader immune–metabolic dysregulation. In silico analyses revealed a significant inverse correlation between cg26053840 methylation and GLO1 mRNA expression in the Broad Institute GDAC Firehose dataset. Genomic annotation further identified putative CEBPD and MYF6 transcription factor binding sites in proximity to the CpG site, suggesting a potential regulatory context. These findings support a model in which glycemic dysregulation increases methylglyoxal production, while reduced renal clearance enhances dicarbonyl stress, potentially driving epigenetic modulation of GLO1. These findings suggest the presence of a metabolic–epigenetic–inflammatory axis, although longitudinal and mechanistic studies are required to determine whether it contributes to organ dysfunction and vulnerability in hospitalized older adults.

## 1. Introduction

Aging is characterized by progressive dysregulation of metabolic homeostasis, low-grade chronic inflammation, and declining renal function, all of which contribute to increased vulnerability to adverse outcomes in older adults [1,2]. Older patients are particularly exposed to both acute and chronic forms of kidney dysfunction, which often co-exist and exacerbate metabolic imbalance [3,4]. Within this context, the “hallmarks of aging” framework provides a unifying view of aging biology, highlighting chronic inflammation, deregulated nutrient sensing, mitochondrial dysfunction, epigenetic alterations, and loss of proteostasis as interconnected drivers of multi-organ decline [5,6]. In particular, metabolic imbalance and renal dysfunction can be interpreted as closely linked manifestations of these mechanisms in older adults, while epigenetic regulation may represent an integrative layer connecting metabolic stress and organ vulnerability in late life. A central biochemical link between hyperglycemia, inflammation, and tissue damage is the accumulation of reactive dicarbonyl compounds, particularly methylglyoxal (MGO), a cytotoxic by-product of glycolysis [7,8]. MGO promotes the formation of advanced glycation end-products (AGEs), leading to oxidative stress, inflammatory activation, endothelial dysfunction, and renal impairment [8,9,10,11,12]. Detoxification of MGO is primarily mediated by the glyoxalase system, composed of glyoxalase 1 (GLO1) and hydroxyacylglutathione hydrolase (HAGH) [8,13]. Despite its key role in metabolic resilience, regulation of this pathway in older patients remains incompletely understood. Chronic kidney disease (CKD), highly prevalent in older adults, is associated with metabolic dysregulation, oxidative stress, and chronic inflammation [14] and is associated with increased carbonyl stress and accumulation of MGO-derived products [15,16]. Experimental and clinical studies consistently show that MGO contributes to renal injury and that MGO-derived adducts increase with declining kidney function and correlate with markers of endothelial dysfunction and inflammation [10,16,17]. Beyond chronic disease, emerging evidence implicates the glyoxalase system in acute kidney injury (AKI) as well. Renal ischemia–reperfusion injury in animal models is associated with a marked reduction in GLO1 activity and a concomitant increase in renal MGO levels, while GLO1 knockdown exacerbates tubular cell death under hypoxia–reoxygenation conditions [18]. Importantly, AKI and CKD are not independent conditions in hospitalized older adults: survivors of AKI face a substantially increased risk of CKD progression. Emerging data further suggest that epigenetic modifications induced by acute ischemic or toxic renal insults may persist long after clinical recovery, a phenomenon termed “hypoxic memory”, and may contribute to the AKI-to-CKD transition [3]. In this context, reduced renal clearance together with increased dicarbonyl production may amplify dependence on glyoxalase-mediated detoxification. At the same time, metabolic dysfunction has been linked to epigenetic remodeling and accelerated biological aging [19,20]. However, epigenetic studies in hospitalized older adults remain limited, and CpG-specific methylation patterns of GLO1 in the context of kidney dysfunction is currently unknown. We therefore investigated CpG-specific DNA methylation within the glyoxal detoxification pathway in hospitalized older adults, focusing on its association with glycemic status, renal function, and systemic inflammation. By integrating epigenetic, metabolic, and clinical data, this study aims to identify molecular signatures linking glycemic dysregulation, inflammation, and kidney dysfunction in late life.

## 2. Materials and Methods

### 2.1. Study Cohort and Data Collection

This study was conducted within the framework of the PROMOTERA study (CE INRCA 20031, 04/02/2021), embedded in the Report-AGE project, a prospective observational study of hospitalized older adults (ClinicalTrials.gov identifier: NCT01397682) [21]. The Report-AGE project enrolled patients aged ≥65 years admitted to acute care wards (geriatric medicine, cardiology, urology, surgery, and neurology) at the IRCCS INRCA hospital in Ancona between June 2012 and February 2019. Inclusion criteria were age ≥65 years, hospital stay ≥24 h, and provision of written informed consent. Patients with hematological malignancies or active COVID-19 infection were excluded. Individuals who died during hospitalization were excluded from the present analysis to reduce bias related to acute clinical conditions. For the present study, patients were included based on the availability of stored blood samples and complete clinical data, including estimated glomerular filtration rate (eGFR), DNA methylation profiles, and inflammatory markers. A total of 271 patients met the inclusion criteria and were included in the final analysis. Participants were stratified into two groups according to kidney function: 193 patients with mildly to moderately decreased renal function (eGFR 45–89 mL/min/1.73 m^2^) and 78 patients with moderately to severely decreased renal function (eGFR < 45 mL/min/1.73 m^2^).

### 2.2. Clinical Assessment and Data Collection Procedures and Ethical Considerations

A comprehensive geriatric assessment (CGA) was performed at admission and discharge using the InterRAI Minimum Data Set for Acute Care (MDS-AC). Blood samples were collected within 24 h of hospital admission in EDTA tubes (Becton, Dickinson and Company, Franklin Lakes, New Jersey, USA) and stored in the INRCA BioGer Biobank. Blood admission and laboratory parameters such as blood cell counts, creatinine, albumin, C-reactive protein, and hemoglobin were measured using standardized procedures. GFR was estimated according to the Chronic Kidney Disease Epidemiology Collaboration (CKD-EPI) equation [22]. Mortality status was obtained from hospital discharge records and regional registries; therefore, no active follow-up was required and no participants were lost to follow-up. Comorbidities were recorded and coded according to the International Classification of Diseases, Ninth Revision (ICD-9): acute myocardial infarction (410–414), congestive heart failure (428), cerebrovascular disease (430–438), dementia (290, 331, 294), depression (296), chronic obstructive pulmonary disease (491, 492, 494, 496), Parkinson’s disease (332), hypertension (401–405), chronic kidney disease (585, 586, 587), anemia (280–285, 790, 6842), diabetes (2500–2507), and cancer (140–172, 174–195, 200–208). The category “dementia” included patients with mild to moderate cognitive impairment who retained decisional capacity and were able to provide informed consent. Multimorbidity was assessed using the Charlson comorbidity index (CCI). Frailty was evaluated using a frailty index based on the deficit accumulation model. The FI included 30 health deficits (e.g., comorbidities, functional impairments, cognitive and sensory deficits, and polypharmacy) and ranged from 0 (no deficits) to 1 (maximum deficits). Participants were categorized as non-frail (FI < 0.10), pre-frail (0.10–0.19), or frail (≥0.20), according to established thresholds [23].

The study was conducted in accordance with the Declaration of Helsinki and approved by the Ethics Committee of the IRCCS INRCA. All participants provided written informed consent prior to enrollment.

### 2.3. DNA Extraction and Methylation Assay

Genomic DNA was extracted from whole blood using the QIAamp DNA Blood Kit (Qi-agen, Hilden, Germeny) and bisulfite-converted using the EZ DNA Methylation Kit (Zymo Research, Irvine, CA, USA). Genome-wide DNA methylation was assessed with the Infinium Human Methylation EPIC BeadChip (Illumina, San Diego, CA, USA), following the manufacturer’s instructions [24]. RStudio software (Version 2023.12.0.369 PBC, Boston, MA, USA) was used for processing and normalization of raw intensities (*. idat) using the R package, Version 2023.12.0.369 PBC, Boston, MA “minfi”[25]. The methylation levels obtained were stored as beta values.

### 2.4. Automated Immunoassay for Chemokine and Cytokine Serum Assessment

Serum levels of interleukin-6 (IL-6), interleukin-17A (IL-17A), growth differentiation factor 15 (GDF-15), cluster of differentiation 163 (CD163), C-X-C motif chemokine ligand 9 (CXCL9), and soluble receptor for advanced glycation end-products (sRAGE) were measured in triplicate using ELLA^TM^ microfluidic immunoassays (ProteinSimple, Bio-Techne, Minneapolis, MN, USA) according to the manufacturer’s specifications. Sensibility was IL-6 (0.11 pg/mL), IL-17A (0.1 pg/mL), GDF15 (0.21 pg/mL), CD163 (3.23 pg/mL), CXCL9 (8.8 pg/mL), and sRAGE (0.82 pg/mL). Intra-assay coefficients of variation (CVs) were all below 10%. The calibration curve for each cartridge is generated by the manufacturer.

### 2.5. Statistical Analysis

Descriptive statistics were used to summarize baseline characteristics, expressed as means ± standard deviations for continuous variables and as frequencies and percentages for categorical variables. The normality of continuous variables was assessed using the Kolmogorov–Smirnov test. Continuous variables not normally distributed were log-transformed prior to analysis. Comparisons between mild-to-moderate and moderate-to-severe kidney function impairment groups were performed using ANCOVA adjusted for age and sex. Categorical variables were compared using chi-square tests. Associations between DNA methylation levels at individual CpG sites within the GLO1 gene and clinical or laboratory parameters were evaluated using linear regression models adjusted for age and sex, diabetes, eGFR, frailty index and CCI. Standardized β coefficients and *p*-values were reported. To account for multiple testing across CpG sites, *p*-values were adjusted using the Benjamini–Hochberg false discovery rate (FDR) method. Specifically, methylation at cg26053840 was tested for associations with fasting glycemia, renal function markers (serum creatinine and eGFR), systemic inflammation markers (CRP, ESR, NLR, and CAR), and circulating cytokines, chemokines, and stress biomarkers (IL-6, IL-17A, GDF-15, CXCL9, CD163, and sRAGE).

Correlation analyses were performed using Pearson or Spearman correlation coefficients, depending on data distribution, to explore relationships between GLO1 methylation and continuous clinical or biomarker variables. A two-sided *p*-value < 0.05 was considered statistically significant. All analyses were performed using SPSS/Win version 29.0.1.0 (171; SPSS Inc., Chicago, IL, USA).

## 3. Results

### 3.1. Clinical Characteristics of the Study Population

Characteristics of the study cohort are presented in Table 1. The patient population consisted of 271 hospitalized older adults enrolled in the PROMOTERA study. Participants were categorized into two groups: 193 with mildly to moderately decreased kidney function (MM group eGFR: 45–89 mL/min/1.73 m^2^) and 78 with moderately to severely decreased kidney function (MS group eGFR < 45 mL/min/1.73 m^2^). Patients with moderately to severely impaired kidney function were significantly older (*p* < 0.001) and exhibited a higher prevalence of ischemic heart disease and dementia (both *p* < 0.05), as well as a greater proportion of frailty (*p* < 0.001). No significant differences were observed between groups for other comorbidities. As shown in Table S1 (Supplementary Materials), the primary causes of hospital admission in the study population were heterogeneous and broadly distributed across clinical categories. The most frequent causes (≥10%) included neurological disorders, particularly neurodegenerative conditions, cerebrovascular complications, and respiratory and gastrointestinal diseases, which represented the leading admission diagnoses in both study groups. Cardiovascular conditions, including chronic ischemic heart disease and heart failure, were also common, although with lower individual frequencies. Overall, no significant differences were observed between the two kidney function groups for most admission categories, except for renal causes, which were significantly more prevalent in MS patients (*p* < 0.01). Laboratory parameters indicated that MS patients had significantly lower red blood cell counts, hemoglobin, and albumin levels compared with those with mildly to moderately reduced kidney function (*p* < 0.01). As expected, renal function markers differed markedly between groups, with higher serum creatinine levels and a lower estimated glomerular filtration rate (eGFR) in the MS group (*p* < 0.001). Inflammatory burden was also increased, as reflected by higher erythrocyte sedimentation rate (ESR) values (*p* < 0.01). No significant differences were observed between groups for the remaining laboratory parameters.

### 3.2. Identification of Glyoxalase Methylation Patterns Associated with Glycemia

To investigate epigenetic regulation of the glyoxal detoxification pathway, CpG-specific DNA methylation within the GLO1 gene was analyzed. Linear regression models adjusted for age, sex, and CCI and estimated proportions of CD4+ T cells, CD8+ T cells, NK cells, monocytes, granulocytes, and B cells were applied to 22 CpG sites (Table 2). The *p*-values were corrected for multiple testing using the Benjamini–Hochberg false discovery rate (FDR). Among these, only cg26053840, located in the 3′ untranslated region (3′UTR), showed a significant association with fasting glycemia, after adjustment for estimated leukocyte proportions derived from DNA methylation data, displaying an inverse relationship (standardized β = −0.295, *p* < 0.01). This association remained consistent across progressively adjusted models including additional clinical covariates (frailty index, ESR, albumin, and RBC count; *p* = 0.027, Table S2, Supplementary Materials). Although the association did not remain significant after multiple testing correction at the CpG level (FDR = 0.066), the consistency of the effect across models suggests that the observed signal is not solely driven by variation in circulating immune cell composition. No other CpG sites within promoter regions (TSS200, TSS1500), gene body, or first exon reached statistical significance, suggesting a site-specific epigenetic association between GLO1 methylation and glycemic control.

### 3.3. Association Between GLO1cg26053840 Methylation and Renal Function

**Figure 1:**
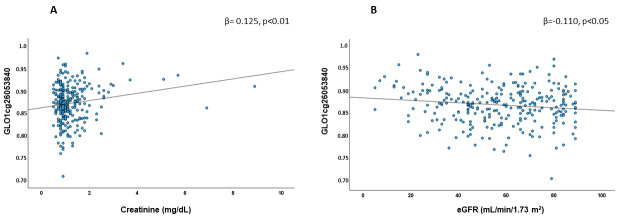
Relationship between GLO1 cg26053840 methylation and renal function parameters. (**A**) Linear regression between GLO1 cg26053840 methylation and serum creatinine. (**B**) Linear regression between GLO1 cg26053840 methylation and estimated glomerular filtration rate (eGFR). Regression lines represent models adjusted for age, sex, diabetes, frailty index, and estimated leukocyte proportions. Beta coefficients and *p*-values are indicated in each panel.

We further explored the relationship between the methylation levels of the identified GLO1 candidate site (cg26053840) and clinical parameters of renal function. Methylation levels at cg26053840 were found to be positively associated with serum creatinine (β = 0.125, *p* = 0.005, Figure 1) after adjustment for age, sex, and diabetes. Conversely, a significant negative correlation was observed between cg26053840 methylation and estimated glomerular filtration rate (eGFR) (β = −0.110, *p* = 0.041, Figure 1), suggesting that higher methylation at this specific site is linked to declining renal function in hospitalized older adults.

### 3.4. GLO1 Methylation and Systemic Inflammation Markers

**Figure 2:**
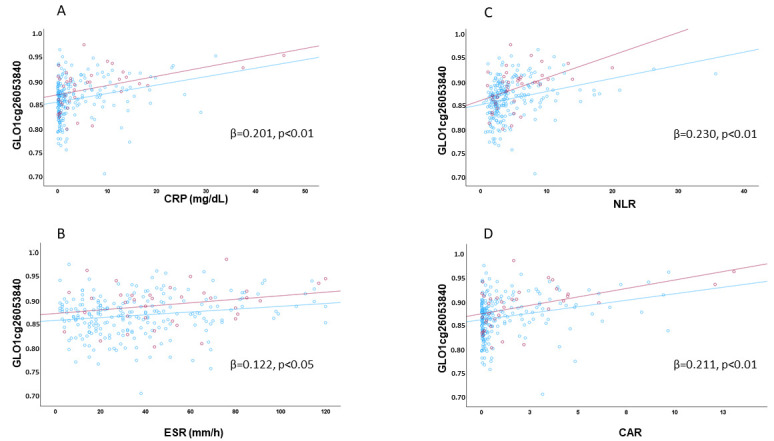
Associations of GLO1 cg26053840 methylation with systemic inflammation indices. (**A**) CRP; (**B**) ESR; (**C**) NLR; (**D**) CRP-to-albumin ratio (CAR). Scatter plots display linear regression models between GLO1 cg26053840 methylation and inflammatory markers. Models were adjusted for age, sex, diabetes, eGFR, frailty index, and estimated leukocyte proportions. Beta coefficients and *p*-values are indicated in each panel. The blue line indicates MM patients, and the red line represents MS patients.

The relationship between GLO1 cg26053840 methylation and systemic inflammation was assessed using linear regression analyses (Figure 2). Higher methylation levels were significantly associated with increased C-reactive protein (β = 0.201, *p* = 0.009) and ESR (β = 0.122, *p* = 0.045).

Similarly, significant positive associations were observed with composite inflammatory indices, including the neutrophil-to-lymphocyte ratio (NLR) (β = 0.230, *p* = 0.002) and the CRP-to-albumin ratio (CAR) (β = 0.211, *p* = 0.001), supporting a link GLO1 cg26053840 methylation and systemic inflammatory burden.

### 3.5. Correlation with Circulating Immune Mediators

**Figure 3:**
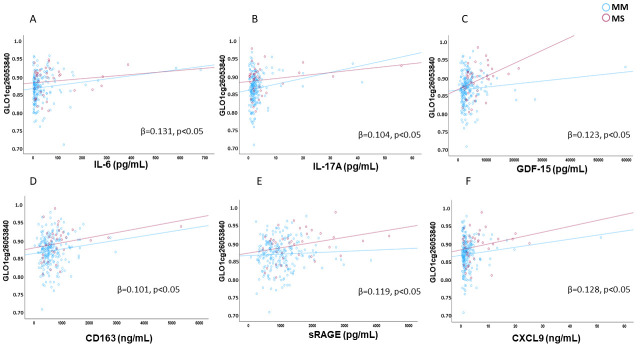
Association between GLO1 cg26053840 methylation and inflammatory and immune mediators (**A**) IL-6; (**B**) IL-17A; (**C**) GDF-15; (**D**) CD163; (**E**) sRAGE; (**F**) CXCL9. Scatter plots display linear regression models between GLO1 cg26053840 methylation and circulating biomarkers. Models were adjusted for age, sex, diabetes, eGFR, frailty index, and estimated leukocyte proportions. Beta coefficients and *p*-values are indicated in each panel. The blue line represents MM patients, and the red line represents MS patients.

Associations between methylation data and circulating immune mediators are shown in Figure 3. Higher methylation at GLO1 cg26053840 was associated with increased levels of pro-inflammatory cytokines IL-6 (β = 0.131, *p* = 0.038) and IL-17A (β = 0.104, *p* = 0.048). Positive associations were also observed with stress- and inflammation-related markers, including GDF-15 (β = 0.123, *p* = 0.044), CXCL9 (β = 0.128, *p* = 0.037), and CD163 (β = 0.101, *p* = 0.045). Additionally, methylation levels correlated positively with soluble RAGE (sRAGE) (β = 0.119, *p* = 0.047), suggesting a potential link with advanced glycation pathways.

### 3.6. Bioinformatic Characterization of the GLO1 cg26053840 Locus

To further explore the potential functional relevance of cg26053840, we interrogated publicly available transcriptomic and QTL databases. Analysis of the Broad Institute GDAC Firehose dataset (https://gdac.broadinstitute.org, accessed on 21 May 2026) revealed a significant inverse correlation between cg26053840 methylation and GLO1 mRNA expression (r = −0.267, *p* = 1.771 × 10^−9^) in prostate adenocarcinoma samples. Interrogation of mQTLdb (http://www.mqtldb.org/cgi-bin/search.cgi, accessed on 21 May 2026) identified predominantly trans-mQTL associations involving variants located on chromosomes 5 and 7, with no evidence of local cis-mQTL regulation on chromosome 6 (Supplementary Table S3). In parallel, analysis of eQTLGen cis-eQTL datasets (https://www.eqtlgen.org/cis-eqtls.html, accessed on 21 May 2026) identified several regulatory SNPs within the GLO1 locus (Supplementary Table S4), although the closest variant was located approximately 866 bp from cg26053840. Examination of the genomic context using the UCSC Genome Browser (hg19) identified putative transcription factor binding sites for CEBPD and MYF6 in close proximity to the CpG site (Figure S1, Supplementary Materials). In addition, previous studies reported cg26053840 as being among the most hypermethylated CpG sites in monocytes from patients with chronic inflammatory diseases, including Behçet’s disease [26].

## 4. Discussion

In this study, we investigated CpG-specific DNA methylation within the glyoxal detoxification pathway in hospitalized older adults with impaired kidney function and its relationship with glycemic status, renal function, and systemic inflammation. We identified a single CpG site within the GLO1 gene (cg26053840) associated with fasting glycemia and further demonstrated links with impaired renal function and increased inflammatory burden. These associations remained significant after adjustment for estimated leukocyte composition, indicating that it is unlikely to be driven by differences in blood cell proportions. Hyperglycemia increases glycolytic flux and the formation of reactive dicarbonyls, particularly MGO, which modifies proteins and nucleic acids through advanced glycation end-product (AGE) formation [8,27,28,29]. The glyoxalase system, primarily driven by GLO1, represents the main detoxification pathway for MGO, and chronic exposure to these species contributes to insulin resistance and diabetic complications, including nephropathy [30,31].

The inverse association between GLO1 cg26053840 methylation and fasting glycemia suggests that methylation levels at this locus may reflect metabolic stress. Although reduced DNA methylation often correlates with increased gene expression, this relationship is context-dependent in non-promoter regions such as the 3′UTR, where methylation may influence transcript stability, microRNA binding, or post-transcriptional regulation [32,33,34]. In silico annotation of this locus identified putative transcription factor binding motifs for CEBPD and MYF6 in close proximity, suggesting a potential regulatory context, although these predictions are based on computational analyses and require experimental validation. DNA methylation has been widely shown to modulate transcription factor binding either by directly interfering with protein–DNA interactions or by recruiting methyl-CpG binding domain proteins [35,36,37]. Although no experimentally validated microRNA binding sites were annotated at this position, DNA methylation within 3′UTR regions has been suggested to indirectly influence post-transcriptional regulation, including RNA stability and alternative processing events [38,39]. Decreased methylation at cg26053840 under hyperglycemic conditions could therefore represent a compensatory response aimed at preserving glyoxalase activity. A significant inverse correlation between cg26053840 methylation and GLO1 mRNA expression was identified in prostate adenocarcinoma samples in the Broad Institute GDAC Firehose dataset [r = −0.267, *p* = 1.771 × 10^−9^ (https://gdac.broadinstitute.org/, accessed on 21 May 2026)], supporting a potential functional link between GLO1 cg26053840 methylation and transcriptional regulation of GLO1. In line with this observation, prior evidence further supports a biologically relevant association between cg26053840 and inflammatory–metabolic regulation, as this CpG site has been reported as being among the most hypermethylated loci in monocytes from patients with chronic inflammatory diseases such as Behçet’s disease [26], consistent with a state of immune–metabolic stress. Experimental evidence supports a bidirectional interaction between dicarbonyl stress and epigenetic regulation, as MGO can modulate DNA methyltransferases, TET (ten–eleven translocation) enzymes, and histone deacetylases [40], while hyperglycemia reduces GLO1 activity [41]. In addition, both diabetes and acute or chronic kidney disease impair the glyoxalase system [42], with reduced GLO1 gene expression and protein levels in advanced CKD [43], suggesting that this compensatory response may be insufficient in advanced disease stages. Consistent with this model, we observed that higher methylation at cg26053840 was associated with impaired renal function, reflected by increased serum creatinine and reduced eGFR. This is in line with the accumulation of carbonyl stress and AGEs characteristic of CKD [16], where reduced renal clearance and increased oxidative metabolism promote systemic dicarbonyl burden and demand for glyoxalase-mediated detoxification [44]. Experimental and clinical evidence further demonstrates that MGO contributes to renal injury, with elevated MG-H1 levels correlating with CKD severity, endothelial dysfunction, and inflammation [10,12,16,17]. In addition, hyperglycemia and MGO induce coordinated transcriptional responses in glomerular endothelial cells and podocytes through their reciprocal interaction [45]. Supporting a mechanistic link with endothelial activation, siRNA-mediated downregulation of GLO1 under hyperglycemic conditions increases VCAM-1 expression in microvascular models [46]. Our findings extend these observations by suggesting that epigenetic regulation of GLO1 may represent an additional layer connecting dicarbonyl stress to renal impairment in older adults. Beyond renal function, GLO1 methylation was associated with multiple inflammatory markers, including CRP, ESR, NLR, and the CRP-to-albumin ratio, supporting a link between glyoxalase pathway regulation and systemic inflammatory burden [17,47,48]. Notably, these associations remained significant after adjustment for estimated leukocyte proportions derived from DNA methylation data, suggesting that the observed signal is not solely explained by major differences in circulating immune cell composition. Nevertheless, given the whole-blood design and the strong interplay among inflammation, renal dysfunction, and metabolic stress in hospitalized older adults, cg26053840 methylation may also reflect a broader systemic epigenetic signature of immune–metabolic dysregulation rather than an isolated locus-specific regulatory event at the GLO1 gene. Associations with IL-6, IL-17A, GDF-15, CXCL9, and CD163 further indicate broad immune–metabolic dysregulation and are consistent with evidence that MGO exerts pro-inflammatory effects on immune cells in type 2 diabetes [49]. In line with this, systemic inflammation has been shown to downregulate GLO1 mRNA expression in vivo [50], suggesting a feedback loop between inflammation and impaired detoxification capacity. The observed association with soluble RAGE is consistent with evidence that MG-H1 upregulates RAGE signaling, promoting oxidative stress, fibrosis, and inflammatory cytokine production in kidney disease [51]. Furthermore, MGO-derived AGEs activate the RAGE/JNK pathway, inducing renal cell apoptosis and mitochondrial dysfunction [52]. Integrating these findings, hyperglycemia increases MGO production, while impaired renal function reduces its clearance, leading to enhanced dicarbonyl stress. In this context, epigenetic modulation of GLO1 may represent either an adaptive or a maladaptive response influencing systemic inflammation and organ dysfunction in vulnerable older adults characterized by multimorbidity. This interpretation aligns with the concept of epigenetic memory in metabolic and renal disease, where sustained exposure to hyperglycemic and hypoxic stress induces persistent epigenetic alterations contributing to long-term organ dysfunction [3]. This includes metabolic memory in diabetes and hypoxic memory in acute kidney injury, involving stable yet potentially reversible changes in DNA methylation and chromatin structure. Accordingly, cg26053840 methylation might represent a molecular imprint of cumulative metabolic–dicarbonyl stress, integrating acute glycemic fluctuations and chronic renal impairment.

Some limitations should be acknowledged. First, the cross-sectional design precludes causal inference, and longitudinal studies are needed to determine whether GLO1 methylation predicts worsening glycemic control, inflammation, or renal decline. Second, DNA methylation was measured in whole blood, which may not fully reflect tissue-specific epigenetic regulation in kidney or metabolic organs. Third, gene expression data were not available, preventing direct evaluation of the functional consequences of cg26053840 methylation. Fourth, although multiple CpG sites within GLO1 were examined, only a single CpG showed significant associations, and replication in independent cohorts is warranted to confirm the robustness of this finding. Fifth, the absence of a control group without renal dysfunction may limit the assessment of the specificity of the observed associations. Sixth, methylglyoxal (MGO) levels were not directly assessed; thus, interpretations regarding MGO-related biology and a potential compensatory response to glycemic status are indirect and require confirmation in future studies. In addition, because the study population consisted of acutely hospitalized older adults, it remains unclear whether methylation at cg26053840 represents a stable epigenetic imprint of chronic metabolic and renal dysfunction or a dynamic response to acute illness and systemic inflammatory stress at the time of hospitalization. Although causes of hospitalization differed across patients, adjustment for all admission diagnoses was not performed because of the heterogeneity and low prevalence of several categories, which could have increased the risk of overfitting and unstable estimates. Overall clinical burden was instead captured through the Charlson comorbidity index and frailty index. Finally, residual confounding from unmeasured factors, including medications, nutritional status, lifestyle factors, and acute illness severity, cannot be excluded. Despite these limitations, this study has several strengths, including the use of CpG-specific epigenetic analysis, comprehensive clinical phenotyping, and integration of metabolic, renal, and inflammatory biomarkers in a well-characterized cohort of hospitalized older adults.

In conclusion, we identified a CpG-specific methylation signature within GLO1 associated with glycemic levels, renal dysfunction, and systemic inflammation. These findings suggest that epigenetic regulation of the glyoxal detoxification pathway may represent a key mechanism linking metabolic dysregulation to inflammation and kidney impairment in aging. Future studies should explore the functional consequences of GLO1 methylation and assess its potential role as a biomarker of metabolic–renal vulnerability and as a target for interventions aimed at reducing dicarbonyl stress in older adults.

## Figures and Tables

**Table 1:** Baseline clinical and laboratory characteristics according to kidney function.

	MM Group N. 193	MS Group N. 78	*p*-Value
Age (years) mean ± SD	82.43 ± 7.1	86.24 ± 5.9	*p* < 0.001
Males n. (%)	81 (42.0%)	40 (51.3%)	0.163
Diabetes n. (%)	67 (34.7%)	36 (46.2%)	0.079
Hypertension n. (%)	115 (59.68%)	42 (53.8%)	0.363
AF n. (%)	37 (19.2%)	17 (21.8%)	0.624
IHD n. (%)	21 (10.9%)	17 (21.8%)	*p* < 0.05
CeVD n. (%)	55 (28.5%)	19 (24.4%)	0.489
COPD n. (%)	28 (14.5%)	13 (16.7%)	0.653
Cancer n. (%)	26 (13.5%)	11 (14.1%)	0.891
Infection n. (%)	55 (28.5%)	24 (30.8%)	0.709
Dementia n. (%)	28 (14.5%)	20 (25.6%)	*p* < 0.05
Frailty index, n (%)			*p* < 0.001
<0.10	49 (25.4%)	6 (7.7%)	
0.10–0.19	74 (38.3%)	26 (33.3%)	
≥0.20	70 (36.3%)	46 (59.0%)	
RBC * (×10^6^/µlL) Mean ± SD	4.20 ± 0.67	3.80 ± 0.57	*p* < 0.001
Hemoglobin * (g/dL) Mean ± SD	12.49 ± 2.07	11.16 ± 1.77	*p* < 0.001
NLR * Mean ± SD	4.52 ± 4.12	5.27 ± 8.20	0.812
Neutrophils * (×10^3^/μL) Mean ± SD	5.47 ± 3.00	6.31 ± 4.38	0.152
Lymphocytes * (×10^3^/μL) Mean ± SD	1.56 ± 0.76	1.50 ± 0.62	0.694
Creatinine * (mg/dL) Mean ± SD	0.92 ± 0.21	2.04 ± 1.24	*p* < 0.001
eGFR * (mL/min/1.73 m^2^) Mean ± SD	67.12 ± 13.16	30.64± 10.32	*p* < 0.001
Fasting glucose * (mg/dL) Mean ± SD	105.30 ± 37.02	108.61 ± 55.06	0.246
ESR * (mm/h) Mean ± SD	35.39 ± 25.09	46.69 ± 30.05	*p* < 0.01
CRP * (mg/dL) Mean ± SD	3.69 ± 5.64	5.27 ± 8.20	0.271
Albumin * (g/dL) Mean ± DS	3.67± 0.55	3.48 ± 0.47	*p* < 0.01

AF, atrial fibrillation; IHD, ischemic heart disease; COPD, chronic obstructive pulmonary disease; CeVD, cerebrovascular disease; RBC, red blood cell; NLR, neutrophil-to-lymphocyte ratio; eGFR, estimated glomerular filtration rate; ESR, erythrocyte sedimentation rate; CRP, C-reactive protein; MM, mildly to moderately decreased kidney function; MS, moderately to severely decreased kidney function. * ANCOVA analysis correcting for age and sex.

**Table 2:** Linear regression analysis of fasting glycemia and DNA methylation levels at GLO1 CpG sites.

CpG Sites	UCSC_RefGene_Group	Standardized Coefficient Beta	*p* -Value	FDR
GLO1cg24164680	Body	−0.094	0.245	0.898
GLO1cg23086892	1stExon; 5′UTR	−0.091	0.184	1.349
GLO1cg13678070	TSS200	0.048	0.466	0.854
GLO1cg27075171	1stExon	−0.036	0.596	0.936
GLO1cg06892152	TSS200	0.002	0.921	1.013
GLO1cg00507815	TSS1500	0.004	0.585	0.990
GLO1cg26053840	3′UTR	−0.295	0.003	0.066
GLO1cg23214328	Body	0.091	0.171	1.881
GLO1cg07249406	Body	−0.075	0.241	1.060
GLO1cg16009289	TSS200	−0.056	0.358	0.875
GLO1cg18089931	TSS1500	−0.026	0.681	0.936
GLO1cg22143547	TSS1500	−0.062	0.385	0.847
GLO1cg26916927	TSS200	0.014	0.854	0.988
GLO1cg07169296	Body	−0.025	0.706	0.862
GLO1cg26824091	Body	−0.006	0.936	0.980
GLO1cg04714954	TSS1500	0.029	0.695	0.899
GLO1cg01498601	Body	−0.037	0.607	0.890
GLO1cg03466367	Body	0.014	0.854	0.988
GLO1cg05967679	Body	−0.127	0.232	1.276
GLO1cg09117841	Body	−0.076	0.313	0.860
GLO1cg24827336	TSS1500	−0.074	0.277	0.870
GLO1cg08713081	Body	0.048	0.452	0.904

The model was adjusted for age, sex, Charlson comorbidity index and estimated proportions of CD4+ T cells, CD8+ T cells, NK cells, monocytes, granulocytes, and B cells and FDR adjusted.

## Data Availability

All data supporting the findings of this study are available within the paper. Other clinical and biochemical datasets used in this study are available from the corresponding author upon reasonable request, subject to compliance with institutional data protection regulations and ethical approval.

## Supplementary Materials

The following supporting information can be downloaded at: https://www.mdpi.com/article/10.3390/life16060917/s1, Table S1. Primary causes of hospital admission in the study population Table S2. Progressive adjustment models evaluating the association between cg26053840 methylation and fasting glycemia after correction for leukocyte composition and clinical covariates Table S3. Genome-wide trans-mQTL associations with cg26053840 (mQTLdb analysis) Figure S1. Putative CEBPD and MYF6 binding sites adjacent to the GLO1 CpG site cg26053840.
