# Supplementary material for: GLO1 cg26053840 Methylation Associates with Kidney Injury and Inflammatory Markers in Hospitalized Older Adults
Source: Life (Basel). 2026 May 29;16(6):917. doi: 10.3390/life16060917 (PMC13302205; doi:10.3390/life16060917)
Supplement: Supplementary file 1 [file life-16-00917-s001.zip › life-4319381-supplementary.pdf]

Table S1. Primary causes of hospital admission in the study population

	MM group<br>N. 193	MS group<br>N. 78	p-value
Cardiovascular / Circulatory disorders n. (%)	9 (4.7%)	4 (5.1%)	NS
Cardiovascular / Chronic ischemic heart disease n. (%)	5 (2.6%)	4 (5.1%)	NS
Cardiovascular / Heart failure n. (%)	7 (3.6%)	3 (3.8%)	NS
Diabetes complications n. (%)	5 (2.6%)	6 (7.7%)	NS
Gastrointestinal / Upper gastrointestinal disorders n. (%)	22 (11.4%)	8 (10.3%)	NS
Genitourinary / Prostatic disorders n. (%)	5 (2.6%)	1 (1.3%)	NS
Sepsis and septic shock n. (%)	1 (0.5%)	1 (1.3%)	NS
Musculoskeletal / Spinal disorders n. (%)	1 (0.5%)	0 (0%)	NS
Neurological / Cerebrovascular complications n. (%)	33 (17.1%)	11 (14.1%)	NS
Neurological / Intracranial hemorrhages n. (%)	6 (3.1%)	0 (0%)	NS
Neurological / Neurodegenerative disorders n. (%)	46 (23.8%)	13 (16.7%)	NS
Renal / chronic kidney complications n. (%)	1 (0.5%)	10 (12.8%)	<0.01
Respiratory pulmonary disorders n. (%)	26 (13.5%)	10 (12.8%)	NS
Severe malnutrition n. (%)	2 (1.0%)	1 (1.3%)	NS
Systemic / Multisystem disorders n. (%)	1 (0.5%)	0 (0%)	NS
Traumatic injury/Fractures of major bones n. (%)	5 (2.6%)	1 (1.3%)	NS
Urinary tract disorders n. (%)	15 (7.8%)	5 (6.4%)	NS
Cancer-related complications n. (%)	3 (1.6%)	0 (0%)	NS

Pearson's chi-squared test p=0.011

Table S2. Progressive adjustment models evaluating the association between cg26053840 methylation and fasting glycemia after correction for leukocyte composition and clinical covariates

Model	Covariates added	β value	p value
Model 1	Age, sex, CCI, Cell-type estimates	-0.266	0.005
Model 2	+ Frailty	-0.269	0.008
Model 3	+ ESR	-0.169	0.009
Model 4	+ Albumin + RBC	-0.224	0.027

Linear regression models were fitted with fasting glycemia as the dependent variable and cg26053840 methylation as the independent variable of interest. Model 1 was adjusted for age, sex, CCI and methylation-derived leukocyte proportions (CD4+ T cells, CD8+ T cells, NK cells, B cells, monocytes, and granulocytes). Subsequent models were progressively adjusted by adding frailty index, ESR, albumin, and RBC count. Each subsequent model included all covariates from the previous model. CCI, Charlson comorbidity index.

Table S3. Genome-wide trans-mQTL associations with cg26053840 (mQTLdb analysis)

Timepoint	SNP	SNP Chr	SNP Pos	A1	A2	MAF	CpG	CpG Chr	CpG Pos	beta	t-stat	Effect Size	p-value	Trans
Adolescence	rs4724942	7	7141565	T	C	0.123	cg26053840	6	38644662	-0.28086	-5.411	0.0063	8.19E-08	Y
Middle Age	rs13167132	5	53379574	G	A	0.013	cg26053840	6	38644662	0.7604	5.6163	0	2.76E-08	Y
Middle Age	rs13189862	5	53380745	A	G	0.013	cg26053840	6	38644662	0.7604	5.6163	0	2.76E-08	Y
Middle Age	rs56264681	5	53381769	T	C	0.013	cg26053840	6	38644662	0.7604	5.6163	0	2.76E-08	Y
Middle Age	rs13190059	5	53380850	A	G	0.013	cg26053840	6	38644662	0.7604	5.6163	0	2.76E-08	Y
Middle Age	rs792019	5	53375731	A	C	0.011	cg26053840	6	38644662	0.83477	5.80777	0	9.40E-09	Y
Middle Age	rs77478573	5	53379153	C	T	0.013	cg26053840	6	38644662	0.7604	5.6163	0	2.76E-08	Y

Data were retrieved from the mQTLdb database (http://www.mqtldb.org/cgi-bin/search.cgi), which catalogs methylation quantitative trait loci across multiple tissues and populations. The analysis was restricted to genetic variants associated with methylation at or near cg26053840 (chr6: 38,644,662; hg19). Only statistically significant mQTL associations reported by the database were included.The column “trans-mQTL (Y/N)” indicates

whether the associated SNP is classified as a trans-acting mQTL according to mQTLdb annotation criteria. All identified regulatory variants are located on chromosomes 5 and 7, whereas cg26053840 is located on chromosome 6, supporting their classification as trans-mQTLs. No cis-mQTLs affecting cg26053840 were identified in the queried dataset.

Figure S1. Putative CEBPD and MYF6 binding sites adjacent to the GLO1 CpG site cg26053840

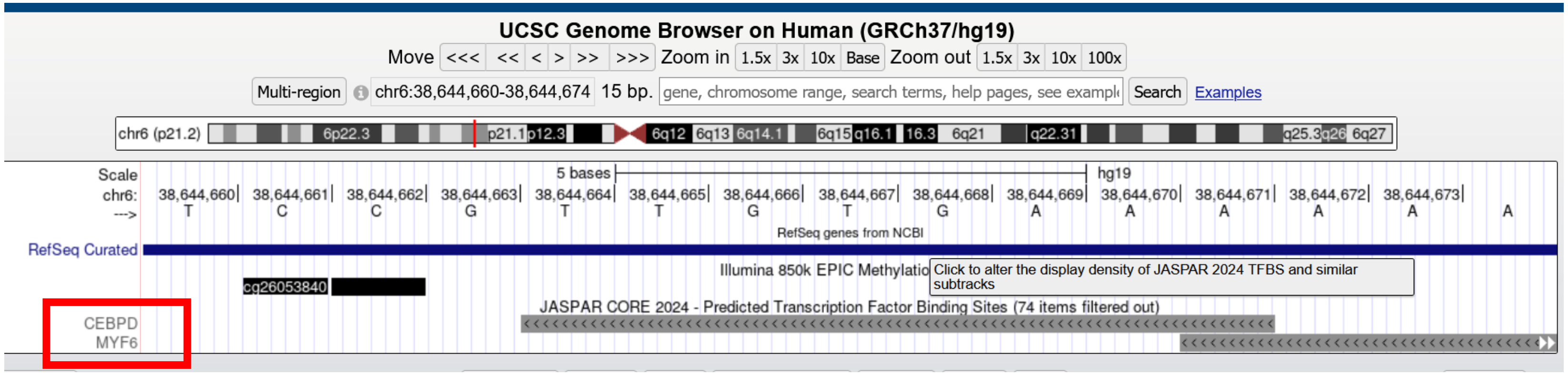

UCSC Genome Browser (hg19) view of the genomic region surrounding cg26053840 within the GLO1 locus. Putative transcription factor binding sites for CEBPD and MYF6 are located in close proximity to the CpG site, suggesting a potential regulatory role of local DNA methylation in transcriptional activity. Coordinates and regulatory annotations were obtained from publicly available UCSC Genome Browser tracks.

Table S4. Mapping of eQTLGen cis-eQTLs for GLO1 relative to cg26053840 genomic position

Pvalue	SNP	SNPChr	SNPPos	AssessedAllele	OtherAllele	Zscore	Gene	GeneSymbol	GeneChr	GenePos	NrCohorts	NrSamples	FDR	BonferroniP	cgPOS	deltaSNP_cgPOS
2.4641E-162	rs7604	6	38643796	T	C	-27.1508	ENSG00000124767	GLO1	6	38657309	36	31569	0	3.1378E-154	38644662	866
2.9165E-162	rs13199033	6	38643471	T	A	-27.1447	ENSG00000124767	GLO1	6	38657309	36	31569	0	3.7139E-154	38644662	1191
4.4066E-126	rs34349982	6	38646114	A	G	-23.8847	ENSG00000124767	GLO1	6	38657309	28	27823	0	5.6114E-118	38644662	1452
1.2416E-162	rs35207097	6	38646235	A	G	-27.1762	ENSG00000124767	GLO1	6	38657309	36	31569	0	1.5811E-154	38644662	1573
1.715E-40	rs937662	6	38646369	T	C	-13.3224	ENSG00000124767	GLO1	6	38657309	36	31569	0	2.1839E-32	38644662	1707
1.3919E-162	rs13212218	6	38646409	A	G	-27.1719	ENSG00000124767	GLO1	6	38657309	36	31569	0	1.7725E-154	38644662	1747
4.3132E-162	rs17544868	6	38642889	C	A	-27.1303	ENSG00000124767	GLO1	6	38657309	36	31569	0	5.4925E-154	38644662	1773
2.2391E-206	rs3799703	6	38646630	A	G	30.6555	ENSG00000124767	GLO1	6	38657309	37	31684	0	2.8513E-198	38644662	1968
1.8046E-40	rs17622097	6	38642371	C	T	-13.3186	ENSG00000124767	GLO1	6	38657309	37	31684	0	2.298E-32	38644662	2291
5.3507E-40	rs9380767	6	38642053	T	C	-13.2372	ENSG00000124767	GLO1	6	38657309	36	31569	0	6.8137E-32	38644662	2609
6.166E-199	rs13216215	6	38647293	T	C	-30.0919	ENSG00000124767	GLO1	6	38657309	36	31567	0	7.8519E-191	38644662	2631
1.4909E-253	rs13213477	6	38641648	A	C	34.0118	ENSG00000124767	GLO1	6	38657309	36	31568	0	1.8985E-245	38644662	3014
3.0615E-163	rs34971977	6	38647810	C	T	-27.2276	ENSG00000124767	GLO1	6	38657309	36	31567	0	3.8986E-155	38644662	3148
4.8854E-27	rs75788409	6	38641476	T	C	-10.7677	ENSG00000124767	GLO1	6	38657309	36	31355	0	6.2212E-19	38644662	3186
1.0725E-40	rs12214815	6	38647852	C	G	-13.3574	ENSG00000124767	GLO1	6	38657309	36	31559	0	1.3657E-32	38644662	3190
3.3738E-124	rs182684016	6	38641355	C	G	-23.7027	ENSG00000124767	GLO1	6	38657309	27	27737	0	4.2963E-116	38644662	3307
5.1321E-248	rs6458065	6	38641354	C	T	33.6353	ENSG00000124767	GLO1	6	38657309	36	31599	0	6.5353E-240	38644662	3308
2.3261E-114	rs140458850	6	38648068	T	C	-22.7287	ENSG00000124767	GLO1	6	38657309	36	31562	0	2.9621E-106	38644662	3406
2.4802E-21	rs116350199	6	38641217	C	T	-9.4824	ENSG00000124767	GLO1	6	38657309	21	21435	0	3.1583E-13	38644662	3445
5.4862E-261	rs1623947	6	38648165	T	C	34.5112	ENSG00000124767	GLO1	6	38657309	36	31556	0	6.9862E-253	38644662	3503
1.0836E-21	rs191404750	6	38648264	C	T	-9.5684	ENSG00000124767	GLO1	6	38657309	21	21435	0	1.3799E-13	38644662	3602
2.4263E-26	rs114762451	6	38648506	G	A	-10.6192	ENSG00000124767	GLO1	6	38657309	33	30680	0	3.0897E-18	38644662	3844

cis-eQTLs for GLO1 were retrieved from the eQTLGen consortium (https://www.eqtlgen.org/cis-eqtls.html). Only variants within ±4 kb of cg26053840 (chr6: 38,644,662; hg19) and with adjusted P-value < 0.05 (BonferroniP) were retained. DeltaSNP_cgPOS indicates the absolute distance (bp) between SNP and CpG position. All coordinates refer to hg19 genome build.
